# The Effect of Brand Internationalization Strategy on Domestic Consumers’ Purchase Intention: Configuration Analysis Based on Brand Authenticity Perspective

**DOI:** 10.3389/fpsyg.2022.891974

**Published:** 2022-06-02

**Authors:** Shaoqing Zhang, Yue Fang, Yuan Zhang, Sihong Zhang

**Affiliations:** ^1^Department of Marketing, Tan Siu Lin Business School, Quanzhou Normal University, Quanzhou, China; ^2^Business School, Department of Human Resource Management, Huaqiao University, Quanzhou, China

**Keywords:** brand internationalization, brand authenticity, configuration analysis, driving path, purchase intention

## Abstract

Brand internationalization is an important strategy for emerging market enterprises to promote their self-owned brand to the international market. It has practical significance for promoting domestic consumers’ trust and acceptance of the international self-owned brands. This paper uses the fuzzy-set qualitative comparative analysis (fsQCA) as the method and 218 consumers in China as the survey subjects. The research focuses on exploring the core factors that stimulate domestic consumers’ purchase intention from the perspective of consumers’ perceived authenticity of brand internationalization (PABI) and how these factors cooperate to affect the driving path of domestic consumers’ high purchase intention. The findings show that (1) country of origin image, quality perception, credibility, and self-identity are the four core factors that stimulate domestic consumers’ purchase intention from the perspective of PABI, but each factor cannot be the necessary condition for high domestic consumers’ purchase intention alone. (2) Three types of conditional configurations constitute the driving path of high domestic consumers’ purchase intention: “country of origin image – self-identity,” “self-identity – credibility,” and “country of origin image – quality perception – credibility.” (3) The potential substitution relationship among the four core factors reflects that emerging market enterprises should choose a targeted driving path to implement brand internationalization strategies; this strategy helps enterprises to enhance domestic consumer trust and acceptance. This study broadens the exploration of brand internationalization through new research methods and perspectives and helps emerging market enterprises to design and implement various targeting, positioning, and segmentation strategies to successfully promote brand internationalization in the contest between globalization and anti-globalization.

## Introduction

In recent years, emerging market consumers’ interest in Chinese global brands has been growing rapidly. In 2021, Google and YouTube’s search index of Chinese global brands has increased by 66% a year in four emerging markets (India, Indonesia, Mexico, and Brazil) (Sina Finance, 2021). It has prompted many Chinese enterprises to actively integrate various marketing methods to build the international image of a self-owned brand and to enhance the consumers’ recognition of Chinese self-owned brands in the international market. However, under the contest between globalization and anti-globalization, Chinese enterprises should allocate resources reasonably to enhance the international image of the self-owned brands and the trust of domestic consumers. This remains a major problem that challenges Chinese enterprises in brand internationalization. In particular, there are differences in the development stage and resource endowments of different enterprises, which require enterprises to effectively identify multiple factors affecting domestic consumers’ purchase intention and its synergistic effects. On this basis, enterprises should choose adaptive reform strategies according to their own factor endowments.

The research on brand internationalization began with scholarly attention to brand names. In a narrow sense, “brand internationalization” refers to the internationalization decision of a brand name, logo, and personality. In a broad sense, “brand internationalization” refers to the process of enterprises establishing brand assets in the international market (Whitelock and Fastoso, 2007). Self-owned brand refers to a brand that is developed by an enterprise with self-owned intellectual property rights. Possessing the powerful capability of self-development and the core technology with a self-owned intelligent right is the most fundamental and key competence for an enterprise (Yu, 2008). Brand internationalization guides consumers to establish strong and unique associations from various aspects, such as product quality, corporate image, and country of origin (Keller, 1998). Brand internationalization plays a significant role in “endorsing” corporate brands. Empirical research shows that brand internationalization changes not only consumers’ recognition of brands in the international market but also domestic consumers’ attitudes toward the self-owned brands (Sheth, 2011), which is highly recognized by domestic consumers (Srivastava and Balaji, 2018). However, these studies did not discuss in depth the reasons for the differences in the purchase intention of domestic consumers. There is scarce research focusing on the analysis of the core factors and their configurations. It could not systematically reveal the complex operation mechanism of multi-factor interaction affecting domestic consumers’ purchase intention.

Purchase intention is a consumer’s awareness or plans to attempt to purchase a brand or product (Ramírez-Correa et al., 2020). The previous study found that the more authentic a brand’s internal and external attributes are perceived by the consumers (e.g., brand quality, process and technology, brand concept, and reputation), the more consumers will trust the brand and generate purchase intentions (Beverland and Farrelly, 2010). However, the increasing commercialization orientation of enterprises undermines the authenticity of the brand (Thompson et al., 2006), which intensifies the doubts and distrust of domestic consumers on enterprises and the internationalization of their brand. As a result, more and more domestic consumers are turning to seek the authenticity of the brand (Kim et al., 2021). It indicates that domestic consumers’ perceived authenticity of an enterprise’s brand internationalization seriously affects their purchase intention toward the brand. Consequently, this paper obtained primary survey data by selecting 218 participants from 17 provinces, autonomous regions, and centrally administered municipalities of China as the survey objects. Using fsQCA, this paper explored how enterprises can coordinate resources to enhance the international image of self-owned brands through “configuration analysis.” Furthermore, we investigate the driving paths that stimulate domestic consumers to increase self-owned brand purchase intentions, as well as the combination of explanatory factors behind different paths. Specifically, this paper will try to answer the following research questions: (1) What are the core factors that stimulate domestic consumers’ purchase intention from the perspective of PABI? (2) How do these core factors work together to influence the purchase intention of domestic consumers? and (3) How many driving paths can arouse the high purchase intention of domestic consumers? Our study makes contributions in the following two aspects. From a theoretical point, this research will help to deepen the rational understanding of the development path and driving mechanism for improving consumers’ purchase intention. From a practical point, it has implications for enterprises to design and implement various targeting, positioning, and segmentation strategies to promote the internationalization process of the self-owned brand.

## Literature Review and Theoretical Framework

### Literature Review

Authenticity is a cornerstone of contemporary marketing practice. There are three forms of authenticity: pure (literal), approximate, and moral (Beverland, 2005; Beverland et al., 2008). Authenticity is a core component of successful brands, which resonates with consumers (Beverland, 2006). Perceived brand authenticity is the authenticity of consumers’ subjective perceptions. Existing research considered it as consumers’ perception and evaluation of brand essence conveyed by brand activities (Schallehn et al., 2014). Consumers pay particular attention to cues that convey authenticity (Beverland and Farrelly, 2010). Carsana and Jolibert (2018) investigated the self-owned brand and found that brand authenticity has a positive impact on brand attitude and purchase intention. Therefore, how to improve consumers’ perceived brand authenticity is regarded as a new marketing method for enterprises to create differential advantages, improve brand value, and increase brand trust (Hernandez-Fernandez and Lewis, 2019).

Internationalization is an effective way to change the attitude of domestic consumers toward the self-owned brand (Song and Shui, 2004). Domestic consumers regard international brands as premium brands since they can successfully meet global needs (Swoboda and Sinning, 2020). Indeed, many self-owned brands perceive brand internationalization as a “springboard” to expand the domestic market and improve their reputation and sales in the domestic market. However, the quality signal, image structure, reputation, social responsibility, standardization, and other brand assets have significantly changed after brand internationalization (Özsomer and Altaras, 2008). The country of origin information has been gradually weakened in the domestic market. The brand commercialization brought by entering the international market will also reduce consumers’ evaluation of the authenticity of the brand (Witek-Hajduk and Grudecka, 2021). In the postmodern market with excessive business and rampant falsehood, authenticity has become the major demand of consumers. Therefore, it is particularly important to systematically explore the attitude and evaluation of domestic consumers on the authenticity elements of a self-owned brand after internationalization.

Safeer et al. (2021b) pointed out that international enterprises can use perceived brand authenticity dimensions (i.e., quality commitment, tradition, and sincerity) as positioning tools to build a real international brand image. Although perceived brand authenticity originates from the internal attributes of the brand, it is reflected in the individual evaluation of consumers (Bruhn et al., 2012). PABI is the subjective evaluation of consumers on the purity of brand internationalization (Napoli et al., 2014). Consumers’ PABI is multidimensional. Bruner (1994) judges authenticity from four aspects: country of origin, quality of production process, credibility, and self-identity. Thus, from the academic point of brand internationalization, this paper will focus on the authenticity elements of consumers’ perception of brand internationalization in four aspects of brand authenticity. Meanwhile, the paper will investigate how these four forms work together to affect domestic consumers’ purchase intention and shape the driving path that generates consumers’ high purchase intention.

#### Country of Origin Image and Consumer Purchase Intention

Country of origin image is the target consumer’s intrinsic impression of a product’s region or country of origin. It is a general perception of the country by consumers (Nguyen and Alcantara, 2022). Country of origin information is a more effective tool for recognizing and evaluating international brands. It positively influences consumers’ intentions to purchase complex products (Hien et al., 2020). After controlling for brand familiarity, perceived brand localness facilitates consumers to form a perception of the authenticity of the brand (Safeer et al., 2021d). Le et al. (2017) explored the relationship between country of origin image and consumers’ purchase behavior and proved that country of origin image has a significant impact on consumers’ perception of product quality, brand attitude, and purchase intention. Berbel-Pineda et al. (2018) found that country of origin image affects not only consumers’ perspective on product quality but also their purchase behavior. Samiee (2019) showed that Asian consumers are increasingly shifting from global brands to domestic brands. Accordingly, the country of origin image will influence consumers’ value evaluation and purchase intention of brands or products, which is one of the important factors to stimulate consumers’ purchase intention.

#### Quality Perception and Consumer Purchase Intention

Quality perception is consumers’ judgment on the quality of purchased products, that is, consumers’ own cognition of product quality. According to the Theory of Reasoned Action (TRA), the judgment of the target object in a certain situation is the key premise affecting the behavioral intention. Perceived product quality, as the antecedent measure of attitude toward the product, will affect consumers’ purchase intention of the product. Calvo-Porral and Lévy-Mangin (2017) explored that customers’ perception of product quality affects customers’ satisfaction and loyalty to products. Safeer et al. (2021a) found that sensory experience positively influenced brand authenticity, which in turn had a significant positive impact on brand affect. Liu et al. (2022) proved that negative quality perceptions limit consumers’ purchase intention. Notably, quality perception is also a considerable factor that cannot be ignored in stimulating consumers’ purchase intention.

#### Credibility and Consumer Purchase Intention

Brand credibility consists of consumers’ perceived beliefs and consumers’ emotional perceptions of the brand, which are derived from consumers’ perceived trustworthiness of corporate commitment (Kang et al., 2021). Credibility is a characteristic of a brand that is worthy of consumer trust. It expresses the direct and explicit cues, such as raw materials, craftsmanship, ideas, and longevity (Molinillo et al., 2022). Credibility factors have a significant impact on people’s purchase intention and behavior. When consumers are uncertain about product attributes and the market is full of asymmetric information, the brand guides consumers as a product positioning signal. Napoli et al. (2014) suggested that a brand’s commitment to quality can build consumer trust, which enhances consumers’ purchase intention. Nayeem et al. (2019) revealed that brand credibility is a potential mechanism by which brand experience affects brand attitude. It proves that a high level of brand credibility can reduce consumers’ uncertainty in purchasing decisions. Moreover, a high level of brand credibility contributes to reducing consumers’ feelings of hesitation toward the brand and enhances consumers’ purchase intention. Therefore, credibility is also a non-negligible part of the influencing factors that stimulate consumers’ purchase intention.

#### Self-Identity and Consumer Purchase Intention

Self-identity is the process and result of consumers’ continuous searching for self-meaning. It is a psychological representation that individuals can both steadily exist and temporarily evoke at the same time (Garbasevschi, 2020). Safeer et al. (2021c) found that sensory and intellectual experience of brand experience positively influences perceived brand authenticity, which can effectively predict consumer loyalty. Consumer behavior toward brands is influenced by identity constructs (Leckie et al., 2021). Brands usually carry “symbolic” symbols, which symbolize consumers’ social status, educational level, and personality traits, among others. Thus, a brand can be considered as a means of building consumers’ identity and status. It reveals that the self-concept connection between consumers and brands is based on the connection between consumers’ unique self-identity and the symbolic meaning of the brand (Malik et al., 2020). Trudel et al. (2016) also found that consumers tend to buy products that can represent the image of the “expected self” and express their identity to others by purchasing or using such products. Therefore, self-identity is also a key factor to stimulate consumers’ purchase intention.

### Theoretical Framework

**FIGURE 1:**
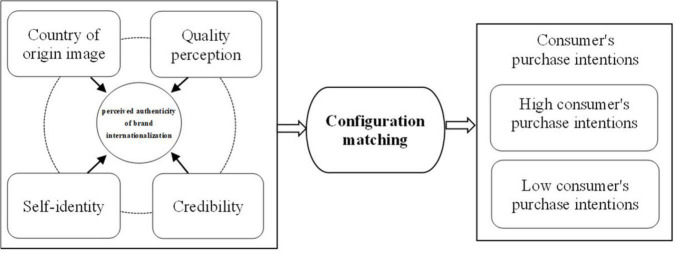
Theoretical framework.

To sum up, from the perspective of consumers’ PABI, the core factors that stimulate domestic consumers’ purchase intention are country of origin image, quality perception, credibility, and self-identity. All these four factors do not affect consumers’ purchase intention independently, but play a synergistic role through linkage matching and generate different driving paths to stimulate high purchase intention of domestic consumers. Therefore, based on TRA and Self-Identification Theory, this paper attempts to build a theoretical model from the perspective of consumers’ PABI. The model will take the domestic consumers’ purchase intention as the outcome variable and country of origin image, quality perception, credibility, and self-identity as the antecedent variables. Using fsQCA, this paper will empirically explore the driving path of brand internationalization strategy to the high purchase intention of domestic consumers through the different “configuration matching” of four antecedent variables, as shown in Figure 1.

## Method and Data

### Fuzzy-Set Qualitative Comparative Analysis

Fuzzy-set qualitative comparative analysis (fsQCA) is a type of qualitative comparative analysis (QCA) that emphasizes the result is produced by the interaction between multiple factors. This method clarifies the multiple paths that produce the result. It aims to analyze the effect of the combination of multiple antecedent variables on the outcomes without considering a causal relationship between the antecedent variable and the result variable (Rihoux and Ragin, 2008). When studying the purchase intention of domestic consumers from the PABI point, the influencing factors often involve many aspects. The change in consumers’ purchase intention under the brand internationalization strategy is the result of the joint action of multiple variables. Therefore, fsQCA is used to carry out empirical tests instead of using the traditional research method. Using the configuration analysis, this paper attempts to explore the complex mechanism of multiple factors that stimulate domestic consumers’ purchase intention of international brands.

### Data

#### Data Collection

A survey was conducted in China, a typical emerging country. The participants should meet two criteria: (1) adult consumers who have lived in China for more than 1 year and (2) those who have more than three times experience in the purchasing of international brands (including). All data were collected via an online electronic questionnaire and an offline random face-to-face questionnaire. A total of 299 questionnaires were collected. After eliminating 81 invalid questionnaires (no experience in purchasing international brands for three times and more than two answers were missing), 218 valid questionnaires were collected (including 31 samples of foreign students in China). The participants come from 17 provinces, autonomous regions, and centrally administered municipalities of China, who have certain knowledge about brand internationalization. Demographic information, such as gender, age, education level, and occupation, is presented in Table 1.

**TABLE 1:** Sample description.

Variable		Number/person	Present/%	Variable		Number/person	Present/%
Gender	Male	100	46	Education level	High school and the lower	24	11
	Female	118	54		College	52	24
	Student	33	15.2		Undergraduate	87	40
	Public officials (government departments, institutions, universities, etc.)	40	18.3		Postgraduate and the higher	55	25
				Age	20 years and the less	9	4.1
Occupation	Enterprise managers	84	38.5		21–30 years	49	22.6
	Enterprise general staff	39	17.9		31–40 years	60	27.5
	Freelancer	18	8.2		41–50 years	71	32.5
	Retiree	1	0.5		51–60 years	21	9.6
	Others	3	1.4		60 years and the greater	8	3.7

#### Variable Measurement

TP-Link (full name: “TP-Link Technologies Co., Ltd.,” Shenzhen, China) is a Chinese brand that supplies computer networking products. It adopted the brand internationalization strategy at the beginning of its establishment. Thus, it is selected as the representative of self-owned brand internationalization in this research. According to the big data of “SpeedTest.cn” (New Wave Business Review, 2021), TP-LINK ranked first in China’s router market share in 2021, which is well known by the majority of Chinese consumers. However, 93.58% of the respondents believe that it is a foreign company, which can effectively avoid the positive answer bias of the country of origin.

From the perspective of consumers’ PABI, this paper takes the country of origin image, quality perception, credibility, and self-identity as the antecedent variables and consumers’ purchase intention as the outcome variable. To ensure the reliability and validity of the scale, the variable measurement and scale were adopted from previous research. All variables were measured with five-point scale items. The specific methods used for the measurement of variables are presented in Table 2.

**TABLE 2:** Scale items, reliability and validity analysis.

Variable	Scale items	Factor loadings	Cronbach’*s*α	AVE	CR	Source
Country of origin image	The technical level of TP-Link in the industry is generally strong	0.821	0.877	0.7	0.88	Han and Terpstra, 1988
	TP-Link is generally well known	0.84				
	The production process and quality of TP-Link are generally good	0.855				
Quality perception	The product quality of TP-Link is very good	0.834	0.918	0.74	0.92	Parasuraman, 1997
	The product of TP-Link are more durable	0.841				
	The product design of TP-Link is highly technological	0.891				
	The product performance of TP-Link is better	0.867				
Credibility	The product quality of TP-Link is trustworthy	0.806	0.895	0.69	0.9	Chaudhuri and Holbrook, 2001
	The product quality of TP-Link is relatively stable	0.866				
	The product quality of TP-Link meets my expectations	0.772				
	I think TP-Link is an honest brand	0.863				
Self-identity	Using TP-Link’s products makes me feel like I’m part of a fashion-conscious group	0.855	0.91	0.72	0.91	Del Rio et al., 2001
	Using TP-Link’s products are personalized	0.821				
	Using TP-Link’s products helps me improve my relationship with the people around me	0.862				
	Using TP-Link’s products will make me look good	0.856				
Purchase intention	I will probably buy TP-Link’s products	0.913	0.924	0.81	0.93	Dodds et al., 1991
	I prefer to buy TP-Link’s products	0.897				
	I will consider buying TP-Link’s products in the future	0.902				
	I would recommend TP-Link’s products to my friends	0.879				

#### Correlation Statistical Analysis

Table 3 presents the correlation coefficient of each variable that affects consumers’ purchase intention. It illustrates that there is a significant correlation between each antecedent variable and purchase intention, which is in line with the expected results.

**TABLE 3:** Correlation statistical analysis.

Variable	Purchase attention	Country of origin image	Quality perception	Credibility	Self-identity
Purchase attention	1				
Country of origin image	0.539**	1			
Quality perception	0.476**	0.328*	1		
Credibility	0.628**	0.426**	0.404**	1	
Self-identity	0.506**	0.483**	0.328**	0.459**	1

n = 218,* indicates p < 0.05; ** indicates p < 0.01, two-sided hypotheses.

#### Reliability and Validity Analysis

The data results of the questionnaire were tested for reliability and validity, as summarized in Table 2. The Cronbach’s α coefficient of each variable is higher than 0.7, which proves that this questionnaire meets the reliability requirements. Using SPSS20 software to calculate the Kaiser-Meyer-Olkin (KMO) value and Bartlett sphericity value, the KMO value is 0.85, which is greater than 0.7, and the Bartlett sphericity test is significant, indicating that the correlation between the items is good. In accordance with Table 2, all the average variance extracted (AVE) values in the samples are greater than 0.5, and all the composite reliability (CR) values are greater than 0.7. Based on the above-mentioned analysis, all the variable item settings of this questionnaire meet the validity requirements.

## Empirical Results and Analysis

### Variable Calibration

Fuzzy-set qualitative comparative analysis is used to explore the ways in which brand internationalization strategy affects domestic consumers’ high purchase intention from the perspective of consumers’ PABI. The original data need to be recalibrated, which is the process of assigning a set affiliation to the cases. According to Coduras et al. (2016), first, based on the data collection of the Likert five-point scale in this paper, the three anchor points of the four conditional variables and one outcome variable are set as the lower quartile, median, and upper quartile of the case data, respectively; second, after determining the fully unaffiliated anchor points, cross-point anchor points, and fully affiliated anchor points, the fsQCA 3.0 software is used to calibrate the original variables so that the transformed pooled affiliation lies between 0 and 1; finally, this is used as the data basis for subsequent fsQCA analysis. The calibration anchor points are listed in Table 4.

**TABLE 4:** Variable measurement and calibration.

Variable	Scale items	Calibration anchor points		
		Fully unaffiliated anchor points	Crosspoint anchor points	Fully affiliated anchor points
Country of origin image	The technical level of TP-Link in the industry is generally strong	3.645	4.000	4.333
	TP-Link is generally well known			
	The production process and quality of TP-Link are generally good			
Quality perception	The product quality of TP-Link is very good	3.657	4.323	4.657
	The product of TP-Link are more durable			
	The product design of TP-Link is highly technological			
	The product performance of TP-Link is better			
Credibility	The product quality of TP-Link is trustworthy	3.333	4.167	4.343
	The product quality of TP-Link is relatively stable			
	The product quality of TP-Link meets my expectations			
	I think TP-Link is an honest brand			
Self-identity	Using TP-Link’s products makes me feel like I’m part of a fashion-conscious group	3.749	4.000	4.501
	Using TP-Link’s products are personalized			
	Using TP-Link’s products helps me improve my relationship with the people around me			
	Using TP-Link’s products will make me look good			
Purchase intention	I will probably buy TP-Link’s products	3.500	4.000	4.443
	I prefer to buy TP-Link’s products			
	I will consider buying TP-Link’s products in the future			
	I would recommend TP-Link’s products to my friends			

### Necessity Analysis

Before the configuration analysis, the necessary conditions of a single variable need to be analyzed, to test the necessity of the four core factors of brand internationalization strategy for the purchase intention of domestic consumers from the perspective of consumers’ PABI. An important indicator to measure the necessary conditions is consistency. It is generally considered that the antecedent condition with consistency greater than 0.9 is a necessary condition. The results of the necessary condition analysis are shown in Table 5, and the consistency of every single condition is lower than 0.9, indicating that any single condition cannot lead to the result of purchase intention. As a consequence, it is necessary to carry out configuration analysis of various paths that generate purchase intention.

**TABLE 5:** Necessity analysis of antecedent variables.

Conditional variable		Outcome variable	
		High consumer’s purchase intentions	Low consumer’s purchase intentions
Brand authenticity	Country of origin image	0.865	0.729
	∼Country of origin image	0.325	0.818
	Quality perception	0.853	0.723
	∼Quality perception	0.330	0.803
	Credibility	0.875	0.739
	∼Credibility	0.269	0.811
	Self-identity	0.865	0.737
	∼Self-identity	0.321	0.797

### Configuration Analysis

In the results of fsQCA, complex, parsimonious, and intermediate solutions are generally obtained. The complex solution only analyzes the actually observed cases and does not use the logical remainder. The parsimonious solution includes all possible logical remainders. The intermediate solution is only based on the logical remainder in line with theory and practice. Generally, compared with complex and parsimonious solutions, scholars prefer to report intermediate solutions and combine parsimonious solutions to distinguish core and edge conditions. The conditions that appear in both the parsimonious and intermediate solution are defined as the core conditions, and the condition that only appears in the intermediate solution is defined as the edge condition.

Referring to the practice of Ragin (2009), this paper sets the original consistency to 0.8 and the frequency to 1 and then combines the PRI consistency greater than 0.7 by using fsQCA3.0. As Table 6 shows, three paths that generate high domestic consumers’ purchase intention are finally obtained.

**TABLE 6:** Configuration analysis of High consumer’s purchase intentions.

Conditional variable	High consumer’s purchase intentions		
	H1	H2	H3
Country of origin image	🌑		🌑
Quality perception			•
Credibility		•	•
Self-identity	🌑	🌑	
Consistency	0.950	0.946	0.951
Original coverage	0.813	0.798	0.691
Unique Coverage Rate	0.022	0.018	0.041
Solution consistency	0.932		
Solution coverage	0.957		

🌑 *Means that the core condition exists, • means that the edge condition exists, “blank” means that the condition may or may not exist.*

Table 6 presents that there are three paths with high purchase intention of domestic consumers, namely, H1, H2, and H3. The overall consistency and consistency of the three paths are 0.950, 0.946, 0.951, and 0.932, respectively, all are higher than 0.9, and the overall coverage rate is 0.957. It indicates that the research model has strong overall explanatory power and high reliability, and the three paths constitute a sufficient condition for high domestic consumers’ purchase intention. The following is an analysis of the three driving paths of high domestic consumers’ purchase intention:

H1: Country of origin image – Self-identify. In this path, country of origin image and self-identify exist as a core condition, and quality perception and credibility may or may not exist. It reflects that once domestic consumers have a good cognition and imagination toward the country of origin of the brand and believe the brand is consistent with their own image (namely, a high degree of self-identity), they will have a high purchase intention. It does not matter whether the product quality perceived by domestic consumers or the trust of international brand influence their high purchase intention. This path is called the “country of origin - self-identity” dominant type, which is the path with the most core conditions among the three paths.

H2: Credibility – Self-identify. In this path, self-identity exists as a core condition, credibility exists as a marginal condition, and country of origin image and quality perception may or may not exist. It shows that the domestic consumers do not care where the brand’s origin is, whether they perceive the quality of the brand’s products, or whether they understand the product’s technology. Once domestic consumers have a great self-identify of the brand and believe the brand is trustworthy, they will be stimulated toward strong purchase intention. This path is called the “self-identity – credibility” dominant type.

H3: Country of origin image – Quality perception – Credibility. In this path, the country of origin image exists as a core condition, the quality perception and credibility exist as marginal conditions, and self-identify may or may not exist. It suggests that domestic consumers mainly rely on identifying the country of origin of the brand to generate purchase intention at this time. Therefore, when domestic consumers have a good stereotype of the country of origin of the international brand, once they think the brand is trustworthy and feel that the products of the brand have good quality and superb technology, they will have a purchase tendency to the brand. This path is called the “country of origin image – quality perception – credibility” dominant type.

## Discussion

### Research Conclusion

With the acceleration of global economic integration and the intensification of international competition, many emerging market enterprises take brand internationalization as their strategic goal to promote their self-owned brand to the international market. However, the core factors and the complex interaction mechanism of brand internationalization strategy affecting the domestic consumers’ high purchase intention have not been discussed in depth. Hence, this paper takes the case of brand internationalization of TP-LINK (a famous enterprise in emerging markets) as a background and uses fsQCA to conduct conditional configuration analysis. The purpose is to explore the core factors that stimulate the purchase intention of domestic consumers from the perspective of consumers’ PABI and how to coordinate the driving path that affects the high purchase intention of domestic consumers.

The research found that first, country of origin image, quality perception, credibility, and self-identity are the four core factors that stimulate the purchase intention of domestic consumers from the perspective of brand internationalization authenticity; however, each factor cannot constitute a necessary condition for the high purchase intention of domestic consumers alone.

Second, three types of conditional configurations constituted the driving path of high domestic consumers’ purchase intention, that is, the dominant path of “country of origin image – self-identity,” “self-identity – credibility,” and “country of origin image – quality perception – credibility.” To elaborate, according to the “country of origin image – self-identify” dominated path, domestic consumers are mainly affected by the country of origin effect and self-identify. Under the combined influence of these two factors, domestic consumers are more willing to purchase self-owned brands. According to the “self-identity – credibility” dominated path, domestic consumers are not only influenced by self-identity but also pay attention to the role of credibility in it. According to the “country of origin effect – quality perception – credibility” dominated path, when the country of origin image becomes the main reason affecting consumers, quality perception and credibility promote domestic consumers to have a high purchase intention toward self-owned brands.

Third, the potential substitution relationship among the country of origin image, quality perception, credibility, and self-identity indicates that the factors affecting domestic consumers’ purchase intention on international brands are multidimensional and complex. According to the building direction and positioning of self-owned brands, emerging market enterprises should choose targeted combination paths to implement brand internationalization strategy, so as to maximize the effect of brand internationalization.

### Theoretical Contributions

The theoretical contributions of this paper mainly focus on the following three aspects:

First, based on TRA and Self-Identification Theory, we constructed a theoretical model and carried out a “configuration analysis” from the perspective of consumers’ PABI. The outcomes indicated the influencing factors and driving path of brand internationalization strategy on domestic consumers’ purchase intention. The existing studies have focused on three aspects: predicting brand attitude from the impact of perceived brand localization and globalization on brand authenticity (Safeer et al., 2021d), predicting consumer behavior from the impact of perceived brand authenticity on brand love (Safeer et al., 2021b), and predicting brand love and customer loyalty from the impact of brand experience on perceived brand authenticity (Safeer et al., 2021a,c). However, existing studies have rarely explored the factors and paths of brand internationalization strategy affecting consumers’ purchase intention from the perspective of PABI. This research focused on the core factors and operation configuration of stimulating domestic consumers’ high purchase intention. The results systematically revealed the complex underlying mechanism of multi-factor interaction affecting domestic consumers’ purchase intention, which not only extended the research of brand authenticity but also broadened the research directions of brand internationalization strategy.

Second, we reviewed the research on the internationalization of self-owned brands in emerging market countries and found that many scholars have proposed lots of useful suggestions for the development of self-owned brand internationalization based on empirical and introspective analysis (Song and Shui, 2004; Zhang, 2015; Swoboda and Sinning, 2020). However, most of them are qualitative research or single-factor causal regression analysis. There is a lack of multi-factor empirical research. With the help of the “configuration perspective,” this paper empirically explored the synergistic effect and linkage matching mode of the four core factors (country of origin effect, quality perception, credibility, and self-identity) affecting consumers’ purchase intention. It filled the gap of empirical analysis on multi-factor configuration matching in the existing research on brand internationalization strategy.

Third, by using fsQCA instead of the traditional statistical regression method, we explored the driving mechanism that stimulates domestic consumers to increase purchase intention toward self-owned brands from the perspective of “causal asymmetry.” The study found the multi-factor configuration with country of origin effect, quality perception, credibility, and self-identity as the core factors can all result in the high purchase intention of domestic consumers differently. This deepens the research on the influence mechanism of consumer purchase intention. Moreover, the findings provided multiple methods for emerging market enterprises to effectively guide domestic consumers to enhance self-owned brand identification and improve their purchase intention.

### Practical Implications

The research conclusions provide three practical implications for emerging market enterprises to implement brand internationalization strategies.

Initially, emerging market enterprises should consider the country of origin effect of their self-owned brand. The driving path reveals that the country of origin image, as a core condition, has a crucial role. To enhance the positive impact of the country of origin effect, we can start with improving the image of the country of origin. First, many consumers have stereotypes about emerging market countries. Emerging market enterprises can cooperate with internationally renowned companies to take advantage of the influence, in order to weaken the stereotypes and make consumers accept domestic self-owned brands easier. Second, emerging market enterprises need to convey self-owned brand culture and product quality to consumers through integrated marketing communications. This changes consumers’ negative stereotypes about self-owned brands in emerging markets. Third, for the purpose of building a superior self-owned brand image with high technology and high quality, emerging market enterprises should increase investment in research and development to improve their own innovation ability and independent research ability continuously and to strengthen the supervision of product quality.

Subsequently, self-identity plays a significant role in influencing consumers’ purchase intention. Self-identity as the core condition occupies two of the three driving paths. The purpose of self-identity is to build a bridge between brands and consumers. First, when emerging market enterprises implement brand internationalization strategies, they should focus on targeting consumers and positioning the self-owned brand, so that the brand image and consumers’ perceptions are closely related. Second, emerging market enterprises should pay attention to the construction of the brand community. A brand community is a network of relationships that consumers associate with based on their shared emotions about a brand (Hsieh et al., 2022). Emerging market enterprises need to deepen consumers’ understanding of self-owned brands through various means and then gradually generate emotions and form a community. Furthermore, emerging market enterprises should guide consumers in the community to actively discuss topics such as user experience and product matching. It helps to improve consumers’ sense of belonging and identification with the self-owned brands.

Ultimately, although quality perception and credibility exist as dependent conditions in the driving path, it does not mean that they are not important. A brand is a credible commitment. First, to win the trust of consumers, the emerging market enterprises must “materialize” trust in a way that consumers can see directly, such as to develop advanced technology, employ high-tech talents, strengthen quality supervision, or improve after-sales service. It makes consumers believe that choosing a self-owned brand is guaranteed. Second, emerging market enterprises should endorse the brand with the help of authoritative institutions, media, and other platforms, to publicize self-owned brands through authoritative platforms to increase consumers’ trust in the self-owned brands. Finally, emerging market enterprises should take after-sales services seriously. As long as emerging market enterprises can give credible commitments to consumers, customers will also repay them with favorable impressions and loyalty.

### Limitations and Future Research

There are still some limitations in this study. First, there is still room for improvement in the division of brand authenticity dimensions. This paper integrates the results divided by scholars and summarizes the dimensions suitable for the characteristics of this study. However, the research perspective and research background are different, and PABI cannot be fully and accurately understood. In future research, it is still necessary to further develop a brand authenticity dimension scale in line with the research background and purpose, which makes the research more targeted. Second, although taking the brand internationalization of TP-LINK (a famous enterprise in emerging markets) as the investigation background is very representative, the product of this brand tends to be more technical. In future research, we should consider designing virtual brands to reduce errors. Finally, due to the availability of data, this paper failed to obtain the survey samples of other emerging market countries, which limits the interpretation of the research conclusions.

## Data Availability Statement

The raw data supporting the conclusions of this article will be made available by the authors, without undue reservation.

## Author Contributions

SQZ and YZ designed and supervised the study. YF collected and analyzed the data. SQZ, YF, YZ, and SHZ wrote the manuscript. All authors contributed equally to this manuscript, reviewed, and approved this manuscript for publication.

## Conflict of Interest

The authors declare that the research was conducted in the absence of any commercial or financial relationships that could be construed as a potential conflict of interest.

## Publisher’s Note

All claims expressed in this article are solely those of the authors and do not necessarily represent those of their affiliated organizations, or those of the publisher, the editors and the reviewers. Any product that may be evaluated in this article, or claim that may be made by its manufacturer, is not guaranteed or endorsed by the publisher.
